# Visualization and Comprehension of Electronic and Topographic Contrasts on Cooperatively Switched Diarylethene-Bridged Ditopic Ligand

**DOI:** 10.3390/nano12081318

**Published:** 2022-04-12

**Authors:** Imen Hnid, Lihao Guan, Elarbi Chatir, Saioa Cobo, Frédéric Lafolet, François Maurel, Jean-Christophe Lacroix, Xiaonan Sun

**Affiliations:** 1 Department of Chemistry, Université de Paris, ITODYS, CNRS, F-75006 Paris, France; im.hnid@gmail.com (I.H.); guanlihao666@gmail.com (L.G.); frederic.lafolet@univ-paris-diderot.fr (F.L.); maurel@u-paris.fr (F.M.); 2 Department of Chemistry, Université Grenoble Alpes, DCM-UMR 5250, F-38000 Grenoble, France; elarbi.chatir@univ-grenoble-alpes.fr (E.C.); saioa.cobo@univ-grenoble-alpes.fr (S.C.)

**Keywords:** photochromic, diarylethene, bipyridine, high-resolution scanning tunneling microscopy, cooperative switches, molecular orbital, DFT calculations

## Abstract

Diarylethene is a prototypical molecular switch that can be reversibly photoisomerized between its open and closed forms. Ligands bpy-DAE-bpy, consisting of a phenyl-diarylethene-phenyl (DAE) central core and bipyridine (bpy) terminal substituents, are able to self-organize. They are investigated by scanning tunneling microscopy at the solid–liquid interface. Upon light irradiation, cooperative photochromic switching of the ligands is recognized down to the submolecular level. The closed isomers show different electron density of states (DOS) contrasts, attributed to the HOMO or LUMO molecular orbitals observed. More importantly, the LUMO images show remarkable differences between the open and closed isomers, attributed to combined topographic and electronic contrasts mainly on the DAE moieties. The electronic contrasts from multiple HOMO or LUMO distributions, combined with topographic distortion of the open or closed DAE, are interpreted by density functional theory (DFT) calculations.

## 1. Introduction

Photoswitchable systems [1], which can be reversibly interconverted between two isomeric states by light irradiation [2], are promising platforms for the design of highly controllable “smart materials” [3,4]. A photochromic molecule adsorbs light at specific wavelengths and thus appears with convertible colors [2,5]. Concomitant with color changes, their physical and chemical properties, such as molecular geometry, dipole moment and electronic structure, alter with isomerization and show switchable on and off states [5,6,7]. The interest in photoswitchable molecules is large and covers diverse research fields ranging from nanomachines (rotaxane-based molecular machines [8,9,10], light-driven rotary molecular motors [11,12]), photoactive nanoparticles (noble metal Nps [13,14,15], fluorescent Nps [16,17]), nanoelectronics [7] (photoactive molecular junctions [18,19,20,21,22], rectifiers [23], memories [24,25] or organic field effect transistors [25,26]), metal organic nanoassemblies (photoswitchable metal-organic molecular cage [27] or metal-organic frameworks [28,29]), supramolecular self-assembled systems (light-driven supramolecular amphiphiles [30], supramolecular polymers [31,32]), biological nanosystems [5,33] and pharmacology [34]. Among all the photochromic molecule families, diarylethene (DAE) [7,35,36], is the most popular on account of its outstanding properties such as: (i) the photochemical switch between the open and closed forms is highly efficient and robust; (ii) both isomers show high thermal stability and fatigue resistance; (iii) upon isomerization, DAE derivatives show significant electronic variations, leading to a HOMO or LUMO frontier orbitals shift, such that the closed form has a reduced energy gap [7,37]. Therefore, the DAEs are excellent stimuli-responsive systems integrating tunable conductivity, electroluminescence, high fatigue resistance and rapid responsibility, which are keys to light up the future nanoworld [1,2,3,4,5].

Once organized on a surface, photochromic switches can be visualized at a molecular level by scanning tunneling microscopy (STM) [10,38,39,40] which always remains challenging. Azobenzene and its derivatives are among the photochromic molecules which have been investigated the most on-surface, and which switch between their cis and trans forms both in ultra-high vacuum (UHV) [41,42,43] and under solid–liquid conditions [44,45,46,47,48]. One drawback of azobenzene derivatives is that, although they are easy to deposit on surfaces, the switch is observed among individual molecules with low efficiency (20–30% switching) [10,38,40,41,42]. Diarylethene derivatives are observed showing highly efficient cooperative switching on surfaces and in solution [36,49,50]. Despite the superior photochromic and electronic properties, they have not been fully investigated and understood by STM at the solid/liquid interface due to the difficulty of depositing and imaging them on a surface in such conditions. Consequently, their photochromism, notably their switchable electronic properties on-surface, has been very rarely investigated down to the submolecular level [49,50,51]. 

Here we explore the photoswitching behavior and the modifications of the electronic properties of a ligand which incorporates diarylethene motif with terminal bipyridine groups. Molecules of bpy-DAE-bpy (DAE = phenyl-diarylethene-phenyl) self-organize upon protonation at the solid–liquid interface and are visualized by STM in both the open and the closed forms with submolecular resolution, such that the local electron densities of states (DOS) can be investigated. First, under ultraviolet (UV) light, the closed bpy-DAE-bpy isomers (CF) with flat π-conjugated DAE cores are organized on-surface. Clear electron DOS contrasts [37,40,43,52,53,54,55,56,57,58,59,60,61] are observed under STM between the multiple highest occupied molecular orbitals (HOMO) and the lowest unoccupied molecular orbitals (LUMO). Second, upon visible or UV light irradiation, the preorganized CF bpy-DAE-bpy isomer switch on-surface; remarkable DOS distinctions between the CF and the open (OF) isomers are demonstrated only on the LUMO images. To the best of our knowledge, switches between DAE CF and OF isomers with such high resolution, giving insight into DOS contrasts, have very rarely been demonstrated previously at a solid–liquid interface. DFT calculations were performed in order to provide interpretations about frontier orbital contributions. 

## 2. Materials and Methods

Synthesis: Syntheses of 1,2-Bis(5-(4′-(4″-[2,2′]bipyridinylphenyl)-2-methylthiophen-3-yl))perfluorocyclopentene(bpy-DAE-bpy). 3,3′-(perfluorocyclopent-1-ene-1,2-diyl)bis(5-bromo-2-methylthiophene) (100 mg, 0.19 mmol, 1 eq), 4-(4-(4,4,5,5-tetramethyl-1,3,2-dioxaborolan-2-yl) phenyl)-2,2′-bipyridine (143 mg, 0.39 mmol, 2.05 eq), K_3_PO_4_ (241 mg, 1.14 mmol, 6 eq) and Pd(PPh_3_)_4_ (21 mg, 0.019 mmol, 10% mol) were dissolved in a degassed 1,4-dioxane/water (5:1) (*v*/*v*) (30 mL) solvent mixture. The solution was heated at 100 °C and stirred for 12 h. After the mixture was cooled to room temperature, it was filtered over celite. The resulting organic layers were washed with water and extracted with dichloromethane (10 mL). The organic phase was dried over anhydrous MgSO_4_ and evaporated to dryness at reduced pressure. The crude product was purified by column chromatography on silica gel using cyclohexane as eluent. After elution of the first fraction, which was the unreacted monobromide derivative, the polarity of the eluent was gradually increased up to cyclohexane/ethyl acetate 50:50 (*v*/*v*), affording 97 mg of the desired compound as a yellow powder (yield: 67%, 0.11 mmol). ^1^H NMR (400 MHz, CDCl_3_) δ 8.78–8.66 (m, 3H), 8.46 (d, J = 7.9 Hz, 1H), 7.85 (td, J = 7.7, 1.8 Hz, 1H), 7.81 (d, J = 8.5 Hz, 2H), 7.68 (d, J = 8.4 Hz, 2H), 7.56 (dd, J = 5.1, 1.9 Hz, 1H), 7.37 (s, 1H), 7.34 (ddd, J = 7.5, 4.8, 1.2 Hz, 1H), 2.04 (s, 3H). ^13^C NMR (100 MHz, CDCl_3_) δ 156.89, 156.17, 149.89, 149.32, 148.47, 142.04, 141.59, 137.73, 137.14, 134.12, 127.88, 127.67, 126.22, 126.17, 124.03, 123.09, 121.46, 121.39, 118.77, 29.84, 14.80. 

STM characterization: Roughly 10^−4^ mol/L solutions of bpy-DAE-bpy in 1-heptanoic acid were prepared. A droplet (20 µL) of these solutions was deposited on a highly oriented pyrolytic graphite (HOPG from Goodfellow) substrate. STM imaging of the samples was performed at the liquid–solid interface by an SPM Nanoscope V (Veeco, Bruker) scanning tunneling microscope at room temperature. Cut Pt/Ir tips were used to obtain constant current images at room temperature with a bias voltage applied to the sample. The STM images were processed and analyzed using the FabViewer application [62]. 

UV-Vis spectroscopy: The UV-Vis absorption spectra of the solution were acquired on a Varian Cary 500 device with tungsten and deuterium lamps, a monochromator and a PbS detector. Solution spectra were obtained by measuring the absorption of bpy-DAE-bpy in 1-heptanoic acid (2 × 10^−5^ M) in a quartz cell with a path length of 1 cm. Pure 1-heptanoic acid was used as a reference solution. The sample was irradiated with a 150 mW Mercury-Xenon Arc Lamp through filters centered at 365 nm and 435 nm for UV and visible light, respectively. Samples were analyzed immediately after irradiation. 

Computations: Ground-state geometry optimizations of monomer, dimer and trimer were performed in the gas phase using density functional theory (DFT) with the ωB97X-D exchange-correlation functional [63] (XCF) and the 6-31G(d) basis set. The use of a long-range dispersion-corrected XCF was employed to accurately describe weak interactions between the chromophores. Basis set superposition error (BSSE) [64,65] between energies of folded and open forms was corrected by means of the counterpoise (CP) scheme [66]. The computed CP corrected energies of interaction were −23.2 and −26.2 kcal mol^−1^ for closed and open trimer forms, respectively (−14.5 and −15.8 kcal mol^−1^ for closed and open dimer forms, respectively). Molecular structures were confirmed as real minima of the potential energy surface on the basis of their harmonic vibrational frequencies, which showed positive force constants for all normal modes. The energies of the molecular orbitals were determined from the optimized geometries of the monomer, dimer and trimer by using the B3LYP functional that is known to give better energies than ωB97X-D.

## 3. Results

**Scheme 1:**
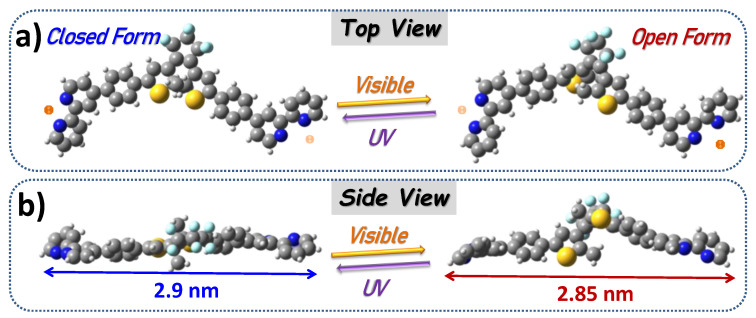
Top (**a**) and side (**b**) views of bpy-DAE-bpy CF and OF isomers switchable by UV-Visible light irradiation. Molecular lengths are calculated. Gray, blue, yellow and white balls represent C, N, S and H, respectively. Orange balls represent H^+^ ions.

The bpy-DAE-bpy ligand is depicted in Scheme 1 with a formula of C_47_H_32_F_6_N_4_S_2_. A series of ditopic ligands (bpy-X-bpy) with variable central bridges has been reported previously by the authors’ group [38,67,68,69]. The bpy-X-bpy building blocks can adopt either the cis-bpy or the trans-bpy configurations, depending on the protonation of the bipyridine units. Here, we employ the bpy-DAE-bpy, where the trans-bpy configuration is generated by bipyridine protonation [38,67,68]. The bpy-X-bpy (trans-bpy) isomers self-assemble with very stable unchanged intermolecular interactions on-surface such that STM characterization is focused on the photochromic switches centered on the DAE units. To study the electronic and chemical switch properties of the DAE cores, highly oriented pyrolytic graphite (HOPG) was chosen as substrate; it provides weak electronic coupling to the molecules in order to avoid the quenching effect [38,70,71]. 

The bpy-DAE-bpy was home-synthesized and was dissolved in 1-heptanoic acid. The solution was irradiated with UV light (365 nm) in order to induce ring closure prior to deposition on the HOPG surface. The CF isomers organization and electronic topography were investigated by STM as an initial reference to compare with the following switched structures. In the second part, the preadsorbed CF self-assembly was irradiated (visible or UV light) on-surface, and photochromic switching between the CF and OF isomers was investigated in situ by STM. 

**Figure 1:**
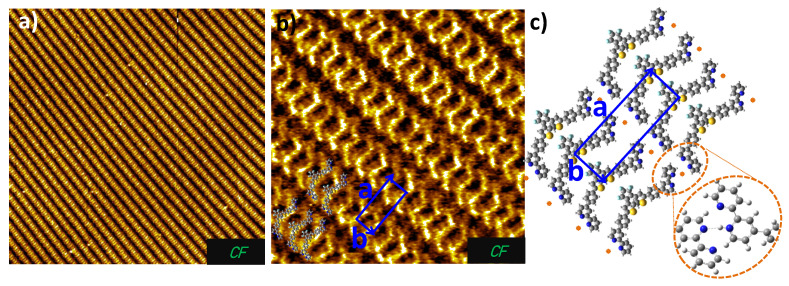
STM images of *CF* bpy-DAE-bpy isomers at 1-heptanoic acid/HOPG interface, I_t_ = 40–50 pA, U_s_ = −0.5 V: (**a**) large-scale: 100 × 100 nm^2^. (**b**) High-resolution: 14 × 14 nm^2^. (**c**) Proposed model of the stripe structure where the orange balls represent protons.

Figure 1 show STM images of the self-assembled CF bpy-DAE-bpy isomers deposited from solutions after UV irradiation (30 min). The overview image in Figure 1a reveals highly ordered two-dimensional (2D) supramolecular stripe architecture. Under STM, full molecules are resolved at submolecular resolution where both the terminal bipyridines and the central DAE cores can be recognized. Molecular structures are superimposed on the STM images in Figure 1b. The bpy-DAE-bpy molecules are observed in the trans-bpy conformation with the ditopic bipyridine groups oriented in opposite directions. The “S”-shaped appearance of bpy-DAE-bpy molecules is in line with our previous studies of bpy-X-bpy derivatives (X = azobenzene, biEDOT or fluorene) [38,67,68,69]. Since the molecules were irradiated in solution with UV, the surface is seen to be overwhelmingly covered by CF isomers under STM (Figure 1b). The unit cell of this network is a parallelogram with cell constants of 3.0 nm (a) and 1.1 nm (b) and an angle of around 82°, as indicated by the blue lines in Figure 1b.

A molecular model using the protonated CF bpy-DAE-bpy is proposed in Figure 1c, where plausible hydrogen bonds between the bipyridines are formed by adjusting the nitrogen positions of the molecules. The protonated CF bpy-DAE-bpy isomers form stable intermolecular N···H-N hydrogen bonds [38,39,40] with adjacent molecules from the neighboring stripes at the two terminal bipyridines in Figure 1c. Each bipyridine unit interacts with its adjacent molecule by double N···H-N hydrogen bonds. There is no direct intermolecular interaction between the CF DAE motifs of adjacent molecules inside one stripe. DFT calculations were performed to verify the existence of hydrogen bonds (inset image in Figure 1c). The details of the DFT calculation are given in the method section. The orientation of the pyridine rings is the result of a balance between the established N···H-N hydrogen bond [53,67,68,69] and a small van der Waals attraction [55,69,72,73,74,75] in the ortho pyridine. As a consequence, the interaction between the two bipyridine rings (each from a neighboring molecule) is very strong (stronger than between two pyridine rings). The intermolecular bonds, together with molecule-substrate van der Waals interactions, stabilize the molecules into parallel stripe patterns [38,67].

STM is a powerful technique which probes the local electron density of states (DOS) [55,56,59,60,61,76] as well as the correlated surface topographic information [40,43,52,53,54,77]. The electron DOS contrast is a complex interplay between the electronic structures of the surface-supported adsorbent (the molecules in our case) and the STM tips [78]. In the STM constant-current mode, the tunneling current depends exponentially on the distance between the two electrodes as well as on the electron DOS from both sides of the tunneling junction, i.e., the tip and the sample [40,43,52,53,54,77,79]. For the same tip in a given lateral position, by varying the bias voltage applied between the tip and the sample, STM visualizes the electron DOS contrasts of the molecules, attributed to different frontier orbitals. In ultra-high vacuum (UHV) environments, the imaging of voltage-dependent DOS has been reported with the metal tip unchanged; for example, the local DOS of C_60_ [55,56]. Phthalocyanine [57,61], pentacene [58], etc. have been extensively imaged at their multiple occupied or unoccupied molecule orbital (MO) states. Alternatively, though difficult, the tip can be functionally modified in UHV to achieve a DOS image. For example, CO- or O_2_-functionalized STM tips have been reported to give more detailed MO images at low temperature [57,58,59,60,61,80,81]. At solid–liquid interfaces, STM tips can be functionalized by adsorbing or desorbing molecules (from the solvent or adsorbent), so that the tip is variable. Ideally, STM is capable of selectively imaging the different frontier orbitals by functionalizing tips without tuning the tunneling conditions. In practice, the observation of electron DOS using functionalized tips is very difficult, particularly at the solid–liquid interface. As a consequence, a clear experimental demonstration and corresponding theoretical interpretations are missing. 

Down to the level of molecular orbitals, pyridine and its derivatives are model systems which can show clear contrasts on the delocalized DOS from the HOMO or LUMO frontier orbitals, especially when forming hydrogen or other molecular bonds [82,83,84]. Regarding the goal of understanding molecular electronic structures, bpy-DAE-bpy is especially interesting with additional photochromic switches where a special separation of the complex occupied and unoccupied molecular orbitals is theoretically predicted [82,83,84].

**Figure 2:**
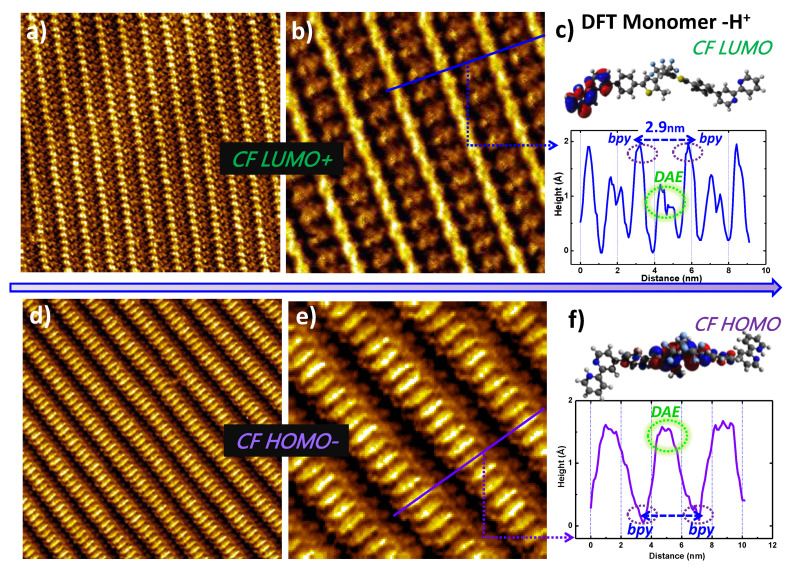
CF bpy-DAE-bpy network at 1-heptanoic acid/HOPG interface, I_t_ = 15~30 pA, U_s_ = −0.45~−0.7 V: STM images of DOS attributed to LUMO orbitals (**a**) 35 × 35 nm^2^; (**b**) 14 × 14 nm^2^. (**c**) Line profile image and DFT-calculated monoprotonated LUMO orbital. Images of HOMO orbitals: (**d**) 35 × 35 nm^2^; (**e**) 14 × 14 nm^2^. (**f**) Line profile image and calculated HOMO orbital.

Under STM, besides the visualization of the full molecules (images in Figure 1), two different types of STM images (Figure 2a,b,d,e) are frequently observed for the CF bpy-DAE-bpy molecules in the same supramolecular self-assembly. In type I images of Figure 2a,b, without affecting the stripe organization, an enhanced DOS is observed to localize at the bipyridine sites whereas the central DAE units appear darker with low DOS (profile image in Figure 2c). On the contrary, in type II images of Figure 2d,e, a reversed DOS contrast is observed. The bipyridine sites appear very dark (almost invisible), whereas the DAE units appear bright and have enhanced DOS (profile image in Figure 2f). To the best of our knowledge, STM images, showing such high resolution with clear DOS contrasts (Figure 2), have never been fully reported at a solid–liquid interface previously. 

To understand the observed electronic contrasts, DFT calculations of the HOMO and LUMO frontier orbitals were performed in the gas phase for monoprotonated CF bpy-DAE-bpy monomers (Figure 2c,d, monomer−H^+^) which simulate the monomer state within the network (with hydrogen bonds at both bipyridines). The calculated LUMO appears mainly on the protonated bipyridine unit (Figure 2c) whereas the HOMO is seen on the phenyl-DAE-phenyl center (Figure 2f). The side-by-side bpy-DAE-bpy stripe organization, either bright on both bipyridines or on the central DAE cores (in Figure 2a,b or Figure 2d,e), resembles that of the LUMO or the HOMO states, respectively. Of course, STM DOS images involve a contribution from multiple orbitals, namely the HOMO- or LUMO+ states, as indicated in Figure 2. More detailed calculations taking into account multiple molecules are necessary to improve the explanation of the DOS contrasts. 

No specific STM tunneling parameters are tuned in order to interconvert between the images in Figure 2 with the reversed electron DOS contrasts, and the alternation between the reversed contrast images is also observed during one image scan (SI Figure S1e). Images resembling the HOMO and the LUMO are observed to alternate, most likely due to spontaneous functionalization (adsorption or desorption of molecules) of the STM tips typical of our experiments at the solid–liquid interface. The pyridine or derivatives have been previously reported to show electron DOS contrasts on the HOMO or LUMO orbitals especially when forming hydrogen bonds [82,83,84], which is identical with what we observe here. 

We can therefore most plausibly interpret this phenomenon as follows: (i) when the Pt/Ir tip images the surface, electrons (holes) tunnel from the tip through the unoccupied (or occupied) surface states, resulting in STM LUMO-resembled (HOMO-) images; (ii) when the organic tip (functionalized by absorption) images, holes (electrons) tunnel from the tip to the occupied (or unoccupied) surface states, leading to STM HOMO-resembled (LUMO-) images. This process is not completely controllable, yet in this experiment, it renders high-resolution image of HOMO and LUMO possible at the solid/liquid interfaces [80,81].

Two key points of the bpy-DAE-bpy DOS images are seen in the comparison of the HOMO and LUMO images in Figure 2. The bpy-DAE-bpy molecules adopt a trans conformation at their bipyridine sites and the strong intermolecular bipyridine-bipyridine hydrogen bonds are identical with those previously observed in bpy-X-bpy self-assemblies, such that the lattice parameter b is always about 1.2 nm (as mentioned in Figure 1c). Moreover, the DAE unit shows a flat symmetric DOS localized at the core center. Although expected, the isomers are confirmed to be in their CF states. 

**Figure 3:**
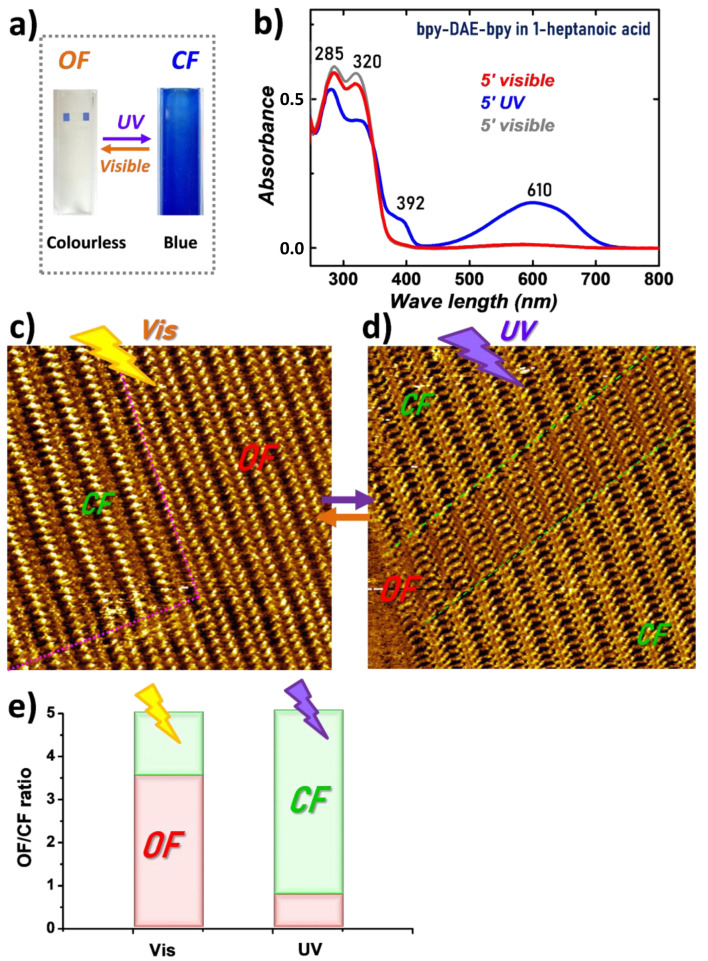
(**a**) Color change from the bpy-DAE-bpy OF and CF isomers, 2 × 10^−4^ M in 1-heptanoic acid, after Vis (λ = 435 nm) or UV (λ = 365 nm) irradiation. (**b**) Absorption spectra with 5 min irradiation upon UV or Vis. (**c**,**d**) STM images of the self-assemblies upon Vis or UV irradiation, respectively: 35 × 35 nm^2^, I_t_ = 15 pA, U_s_ = −0.55 V. (**e**) CF/OF ratio after irradiation estimated from STM images.

The photochromism of bpy-DAE-bpy molecule was investigated both in solution by UV-Vis absorption spectroscopy and on-surface by STM (Figure 3). The difference in the colors of the OF (colorless under Vis, λ = 435 nm) and the CF (blue under UV, λ = 365 nm), produced by irradiation of 1-heptanoic acid solutions, is visible to the naked eye (Figure 3a). Figure 3b shows the changes in the absorption spectra upon irradiation. The initial UV-Vis absorption spectrum of the solution irradiated by visible light presents two characteristic bands at 285 nm and 320 nm (red curve in Figure 3b), attributed to the bipyridine groups and the central DAE cores, respectively. This spectrum does not show any peak in the visible range, which confirms that all the molecules are in the open form, OF; the solution is then colorless. Next, the solution was irradiated with UV light for 5 min (blue curve in Figure 3b). The spectrum obtained displays two new peaks: a sharper one at 392 nm and a broader one centered at around 610 nm, which is consistent with the blue color observed. This drastic change after UV irradiation clearly shows the photoconversion of bpy-DAE-bpy between OF and CF. Then, the solution was irradiated again with visible light (gray curve in Figure 3b). The initial spectral characteristics were completely recovered. This confirms the reversibility of the isomerization of bpy-DAE-bpy, which is typical of diarylethene derivatives [18,85].

STM was employed to investigate the molecular photochromism upon irradiation in situ at the solid–liquid interface. Solutions containing bpy-DAE-bpy (both OF and CF) were first deposited on HOPG and then irradiated. The STM image in Figure 3c was recorded after 10 min exposure to visible light (435 nm) where both the OF and CF isomers are seen to coexist in a single domain. The separation between the different CF/OF forms is marked by the pink dashed lines. The CF isomers attributed from their LUMO- orbitals (Figure 3c, left) can be recognized, where the bipyridine sites appear brighter and the central DAE cores appear darker (same as in Figure 2a,b). A different kind of STM image, containing an extra-bright center between the terminal bipyridines (Figure 3c, right), is therefore suggested to be the OF isomer. From the STM images obtained after visible (435 nm) light irradiation, the OF isomers are in the majority (Figure 3e). The STM image in Figure 3d was recorded after a 10 min UV irradiation (365 nm) in situ of molecules deposited on the surface. As expected, mainly CF isomers are now observed. The molecular switching can take place in combined environments, in the liquid phase and on top of the surface, where we defined it as a switching at the solid–liquid interface. 

In all STM images of the surfaces for various irradiation time, the bpy-DAE-bpy OF and CF isomers coexist (within a single domain or in different domains), but do not mix individually, contrary to those from bpy-azo-bpy molecular switches [38]. The supramolecular stripe organizations are maintained unchanged despite the CF/OF switching, such that neither domain separations nor defects appear at the boundary (dashed lines in Figure 3c,d) between the switched isomers. According to our analysis of the STM images, it is easier to stabilize the CF forms on-surface after UV irradiation; Figure 3e and Figure S2 demonstrate that the CF isomers can reach 80% from the total molecules on surface. The OF isomers can reach about 60% surface occupation after visible light irradiation. The isomers are able to switch (though not 100%) between their open and closed forms on the surface. The alternation between the predominant CF or OF forms upon irradiation demonstrates clear cooperative photochromic switches at the solid–liquid interface. This suggests that some interactions between the two adjacent DAE open rings exist which probably lead to the observed cooperative switches on-surface [49,50]. Our next objective is to achieve extremely high-resolution STM images (as those shown in Figure 2) which allow an insight into the electronic or chemical modification generated from the isomeric switches. 

**Figure 4:**
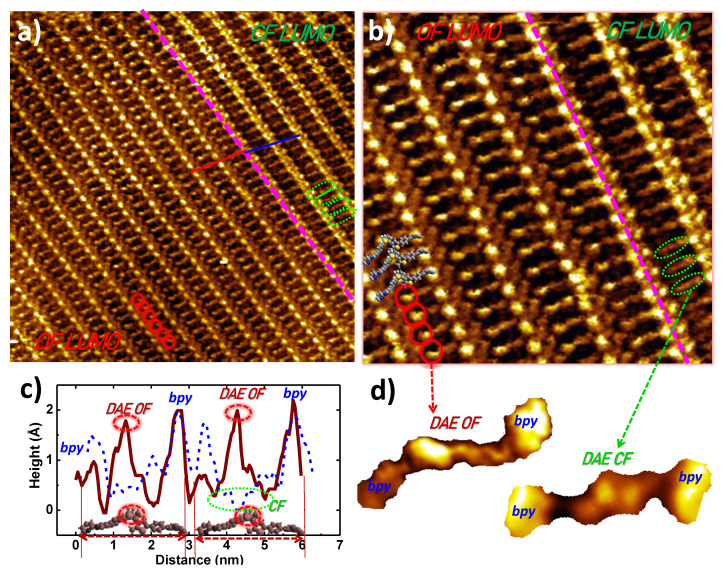
STM LUMO images of bpy-DAE-bpy show both the CF and the OF isomers at the 1-heptanoic acid/HOPG interface. Red circles: DAE cores in OF; green circles: CF. Tunneling parameters: I_t_ = 20 pA, U_s_ = −0.5~−0.7 V (**a**) 35 × 35 nm^2^. (**b**) High-resolution: 15 × 15 nm^2^. (**c**) Line profiles of red and blue lines from image in (**a**) corresponding to OF and CF isomers, respectively. (**d**) Images of single OF and CF isomers extracted from image (**b**) for a clear view of the electron DOS.

In Figure 4a, bpy-DAE-bpy molecules exist in both the OF and the CF forms on-surface. On the right-hand side of the dashed pink line, an electron DOS contrast is observed where the bipyridine sites are bright and the central phenyl-DAE-phenyl units appear darker, corresponding to the CF LUMO image (as demonstrated in Figure 2). A single-molecule image of the CF LUMO is extracted (Figure 4d, right). The phenyl-DAE-phenyl backbone appears flat and its DOS has a symmetric triangular shape representing the closed DAE ring. Taking this CF LUMO image as a known reference, the left-hand side of the image shows completely different electronic contrast: does this represent the OF isomer? The bpy-DAE-bpy OF structure is superimposed onto the high-resolution STM image (Figure 4b) where single molecules are identified. The single-molecule image is extracted (Figure 4d, left inset). At a submolecular level, the two terminal bipyridines (from the same bpy-DAE-bpy molecule) show delocalized DOS, such that the bipyridine on the left appears dark and the one on the right appears bright; this arises most probably from an electronic contrast. The phenyl-DAE-phenyl backbone appears unsymmetrical, with the left-hand side dark and the right-hand side bright. This strongly suggests that the images represent the OF isomer, as such contrast can arise from the DAE open-ring distortion, namely a topographic contrast, while electronic contrast may also play an important role. A line profile image across the two different OF and CF isomers in Figure 4a is shown in Figure 4c, where the profiles are marked in solid red and dashed blue lines, respectively. The phenyl-DAE-phenyl backbone is lower in the CF and higher in the OF, confirming a CF-to-OF switch at the DAE core. The molecules are clearly distinguished as their CF or OF isomers under STM (Figure 4d). The topographic as well as the electronic contrasts between the OF or CF forms are expected of the phenyl-DAE-phenyl units due to the open/closed DAE ring distortion. Whereas the DOS variation from the molecule CF to OF is surprisingly observed on the bipyridine sites. The OF observed in Figure 4 resembles the LUMO image in Figure 2b. The distinction between the OF and CF forms is therefore observed most probably from the LUMO images. To our surprise, coexistence of the two isomers has never been observed for the HOMO images (as those shown in Figure 2d,e). 

**Figure 5:**
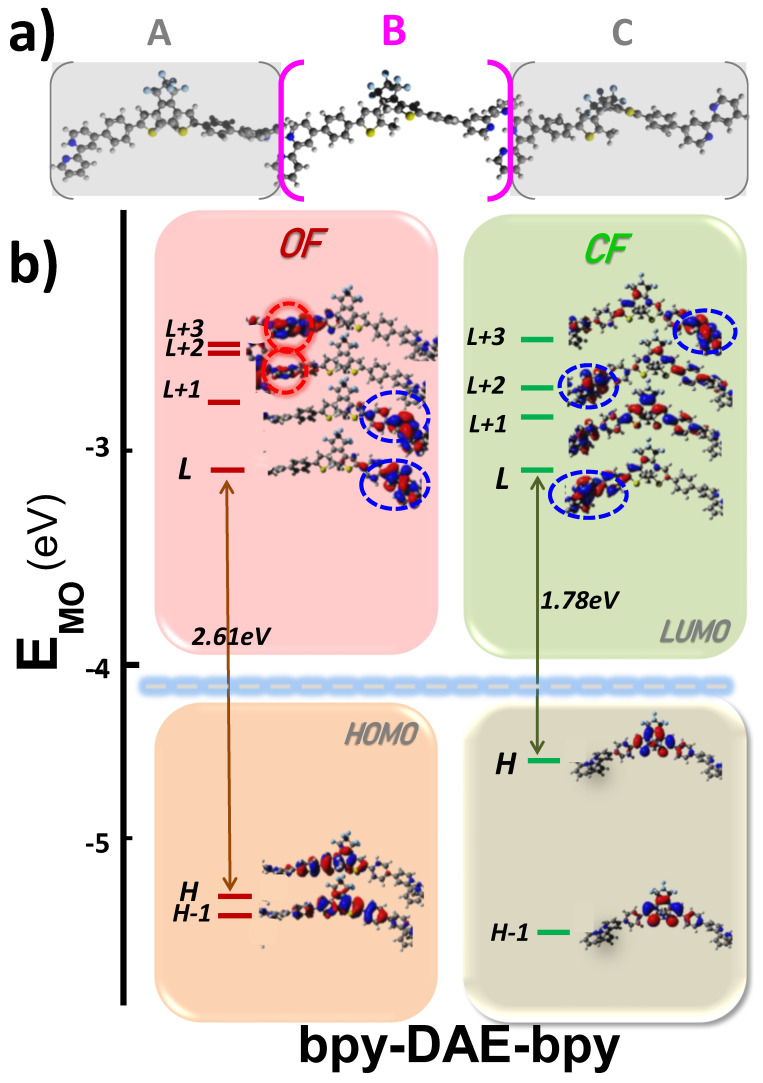
(**a**) DFT calculation for protonated bpy-DAE-bpy trimers, marked as A, B and C. (**b**) Frontier MOs of CF and OF from the B molecule in (**a**) are shown at different energy levels. Blue and red dashed circles highlight the enhanced orbitals from either the bipyridine or the phenyl-DAE-phenyl units, respectively. E_F_ is here defined from the known HOPG Fermi level [84].

In order to obtain a better understanding of the electron DOS delocalization owing to the different photochromic forms of the isomers, more DFT calculations were performed to determine the orbital energies of protonated bpy-DAE-bpy trimers in the gas phase. In this case, the middle monomer, marked B (Figure 5a), has almost identical intermolecular interactions as those from inside the self-organized supramolecular stripes. Calculated multiple LUMO and HOMO frontier orbitals of the B unit, from both the OF and CF isomers, are shown in Figure 5b. Note that the relative values of the graphite Fermi level and of the HOMO and LUMO of the DAE in Figure 5 are not quantitative, as the DAE HOMO and LUMO values are calculated in the gas phase where the DAE/HOPG interactions have not been taken into account.

For the LUMOs, the CF bpy-DAE-bpy isomers have a low band gap around 1.78 eV and yield the frontier orbitals located on both the protonated left (LUMO and L + 2) and the neutral right bipyridine sites (L + 3). The OF isomers appear to be different, and have a broader band gap around 2.61 eV, with the frontier orbitals located at the right bipyridine (LUMO and L + 1) and the DAE left “arm” (L + 2 and L + 3) [37]. STM can visualize these LUMO orbitals under reasonable tunneling conditions which correspond to unsymmetrical DOS contrasts on the phenyl-DAE-phenyl open ring, as indicated by the red circles in Figure 5b. We therefore conclude that the OF phenyl-DAE-phenyl unit, showing bright unsymmetrical DOS, can be attributed most probably to combined LUMO electronic and topographic contrasts. The OF bpy-DAE-bpy also appears with a DOS delocalization mainly on the bipyridines where the molecular orbitals emerge mainly on the right bipyridine in Figure 5b. Moreover, the calculated LUMO frontier orbitals from the CF or the OF are rather close to the HOPG Fermi level [86] (E_F_ in Figure 5b), and are almost consistent in their energy levels. The value of E_F_ is simply marked as a reference here according to the HOPG Fermi level because the precise E_F_ of the DAE is beyond the calculation capability. These explain why we observed both isomers in the same LUMO STM image using the same tunneling conditions, whereas the CF and the OF appear with clear DOS contrasts due to the DAE switch in Figure 4.

The calculated HOMOs yield their frontier orbitals centered at the phenyl-DAE-phenyl backbones for both the CF and the OF isomers. Contrary to the rather similar LUMO energy levels from the two forms, the CF and the OF HOMOs have very different energies (Figure 5b). The CF yields the HOMO frontier orbital close to the E_F_ whereas the OF HOMO is remote. Consequently, we have never observed both isomers in the same HOMO image simultaneously, nor can we visualize the OF by the HOMO images. Such high-resolution images are essential to distinguish the DAE OF or CF forms from their electronic DOS variation. The adjacent DAE-DAE open-ring interactions inside the organized stripes, which appear only in their open forms, are likely at the origin of the DAE cooperative switches observed by many groups previously. 

## 4. Conclusions

In summary, a novel diarylethene derivative bpy-DAE-bpy has been synthesized for self-assembly at the solid–liquid interface and is investigated by STM at extremely high submolecular resolution. The bpy-DAE-bpy molecules were first UV-irradiated in solution and stabilized on-surface overwhelmingly in the closed form. The CF isomer, which is usually known as its metastable form [21,87], immerses with high stability at the liquid/HOPG interface. Clear electronic contrasts are observed between the CF LUMO and HOMO images, thanks to functionalization of the tip whose electronic structure varies from the decoration of organic absorbents. The CF LUMOs show enhanced DOS located on the bipyridine sites whereas the HOMOs show reversed DOS on the phenyl-DAE-phenyl backbones. Investigation of on-surface photochromism reveals cooperative CF or OF switching upon light irradiation. Most importantly, a clear distinction between the CF and the OF is observed, down to a submolecular level, merely on the LUMO images. Different from the flat DOS of the CF isomer, the OF phenyl-DAE-phenyl backbones show unsymmetrical shapes attributed to both topographic and electronic LUMO contrasts. DFT calculations provide an insightful explanation of the electronic DOS contrasts from the molecule on its HOMO or LUMO, and on its open or closed forms. Such visualization of the bpy-DAE-bpy switches down to the DOS level is demonstrated for the first time by STM at the solid–liquid interface which provides deep understanding of electronic as well as topographic properties of diarylethene photochromism on surfaces. 

## Data Availability

Not applicable.

## Supplementary Materials

The following supporting information can be downloaded at: https://www.mdpi.com/article/10.3390/nano12081318/s1, Figures S1–S3: STM results; Figure S4: OF/CF Switching ratio; Figures S5–S11: Calculation.
