# Supplementary material for: Visualization and Comprehension of Electronic and Topographic Contrasts on Cooperatively Switched Diarylethene-Bridged Ditopic Ligand
Source: Nanomaterials (Basel). 2022 Apr 12;12(8):1318. doi: 10.3390/nano12081318 (PMC9029802; doi:10.3390/nano12081318)
Supplement: Supplementary file 1 [file nanomaterials-12-01318-s001.zip › nanomaterials-1629699-supplementary.pdf]

Supporting Information

Visualization and Comprehension of Electronic and Topographic Contrasts on Cooperatively Switched Diarylethene-Bridged Ditopic Ligand

Imen Hnid [1], Lihao Guan [1], Elarbi Chatir [2], Saioa Cobo [2], Frédéric Lafolet [1], François Maurel [1], Jean-Christophe Lacroix [1,*] and Xiaonan Sun [1,*]

[1] Department of Chemistry, Université de Paris, ITODYS, CNRS, F-75006 Paris, France; im.hnid@gmail.com (I.H.); guanlihao666@gmail.com (L.G.); frederic.lafolet@univ-paris-diderot.fr (F.L.); maurel@u-paris.fr (F.M.)

[2] Department of Chemistry, Université Grenoble Alpes, DCM-UMR 5250, F-38000 Grenoble, France;
elarbi.chatir@univ-grenoble-alpes.fr (E.C.); saioa.cobo@univ-grenoble-alpes.fr (S.C.)

* Correspondence: lacroix@u-paris.fr (J.-C.L.); sun.xiaonan@u-paris.fr (X.S.)

1. STM results p. 2–4

2. OF/CF Switching ratio p. 5

3. Calculation p. 6–12

1. STM results

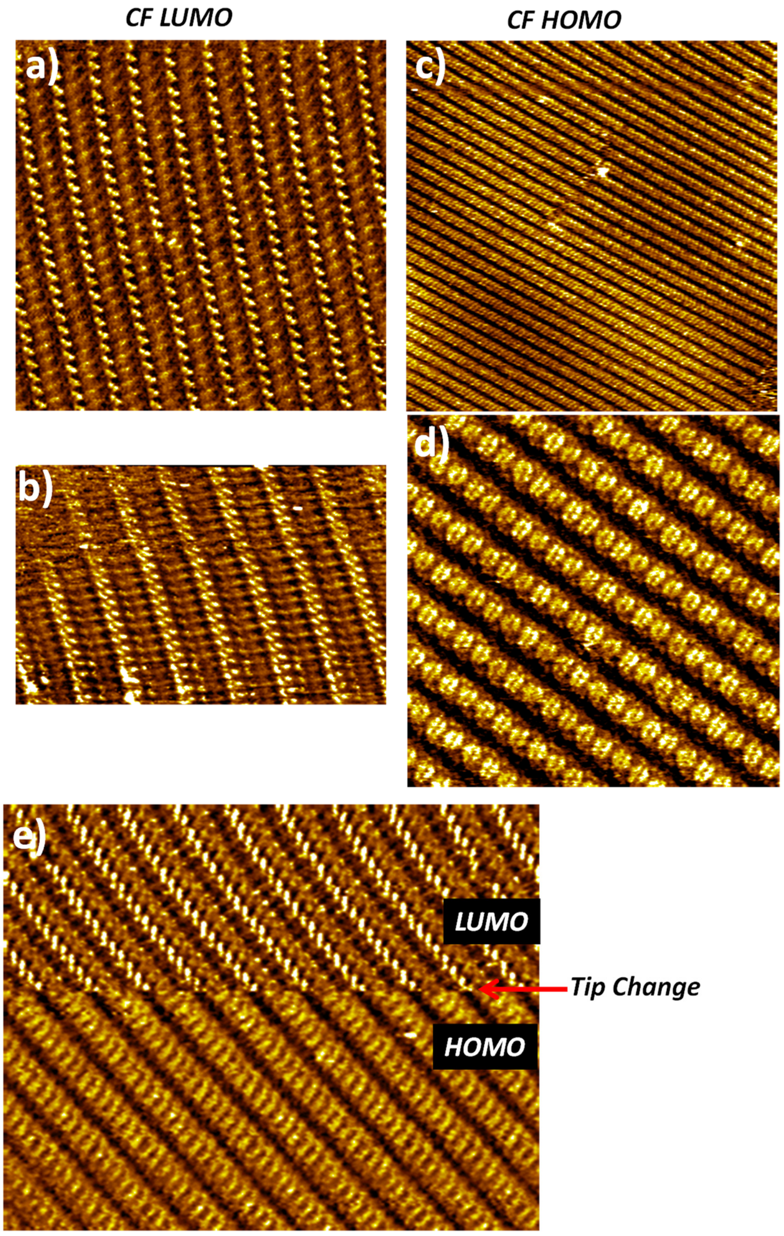

Figure S1. STM images after UV irradiation on bpy-DAE-bpy trans-like organization at the 1-hetanoic acid/HOPG interface. CF isomers are observed in their LUMO images (a) 25×25 nm^2, (b) 28×18 nm^2 I_t = 15-25 pA, U_s = -0.6 V. CF isomers are observed in their HOMO images, (c) 80×80nm^2 (d) 22×22nm^2 (e) 40×32nm^2 I_t = 15-25 pA, U_s = -0.5~-0.7 V.

The bpy-DAE-bpy molecules stabilize overwhelmingly as CF isomers after UV irradiation. The different STM images with clear electronic contrasts from the LUMO and HOMO are shown in Figure. S1 a to d, respectively. Figure. S1 e records an image where the STM tip changes (desorb/resorb molecules) during the scan, so that the top part of the image indicates a LUMO topography whereas the bottom part indicates a HOMO topography.

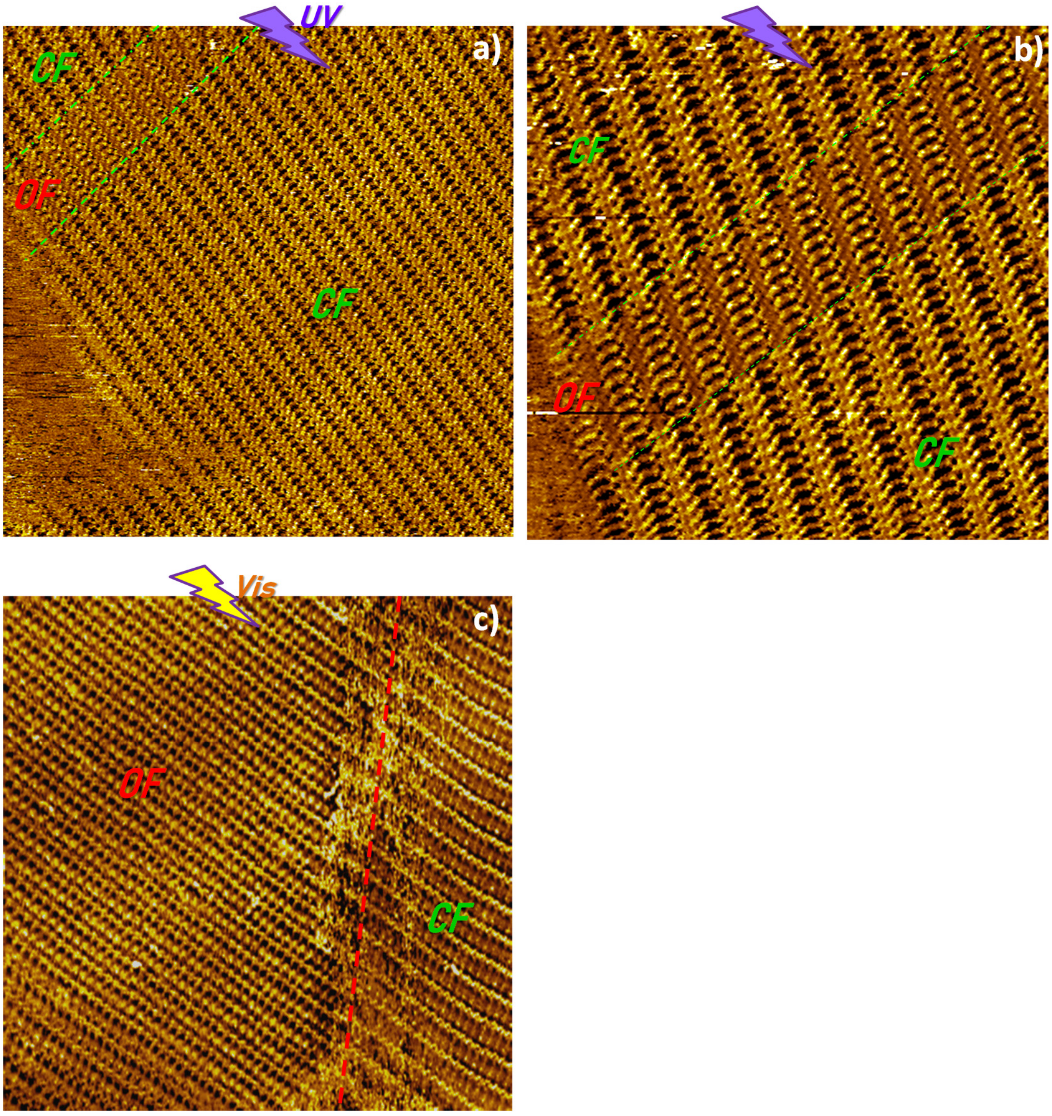

Figure S2. STM LUMO images of bpy-DAE-bpy organization with mixed CF and OF isomers. After UV irradiation: (a) 80×80nm^2 (b) 35×35nm^2, I_t = 15pA, U_s = -0.55V. After Vis irradiation: (c) 50×50nm^2, I_t = 20pA, U_s = -0.5V.

The bpy-DAE-bpy molecules stabilize as mainly CF isomers after UV irradiation whereas small stripe of OF isomers organizations are visible (Figure S2 a and b). The bpy-DAE-bpy molecules stabilize as mainly OF isomers after Vis irradiation whereas a small domain of CF isomers organizations can still be observed (Figure S2 c).

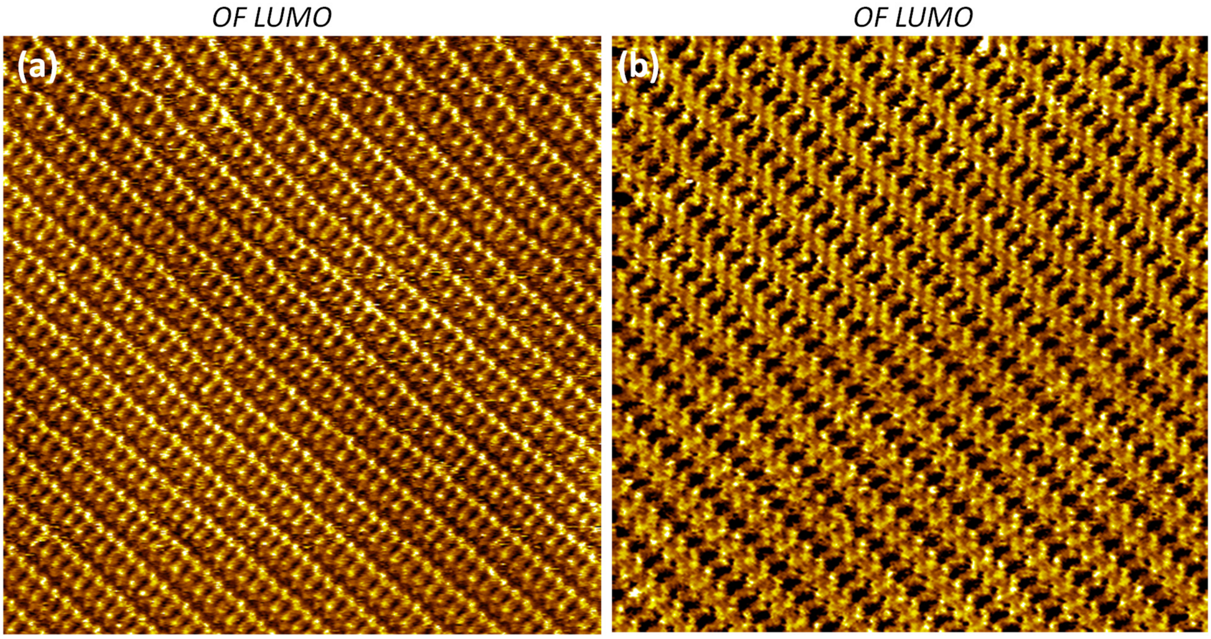

Figure S3. STM LUMO images of bpy-DAE-bpy organization with OF isomers. After 5 minute visible irradiation in solution and then deposition on HOPG: (a) 38×38nm^2 (b) 32×32nm^2, I_t = 20pA, U_s = -0.5V.

The bpy-DAE-bpy in solution is first exposed to visible irradiation and then deposited on HOPG. The molecules are observed as CF isomers (Figure S3 a and b).

2. **OF/CF Switching ratio**

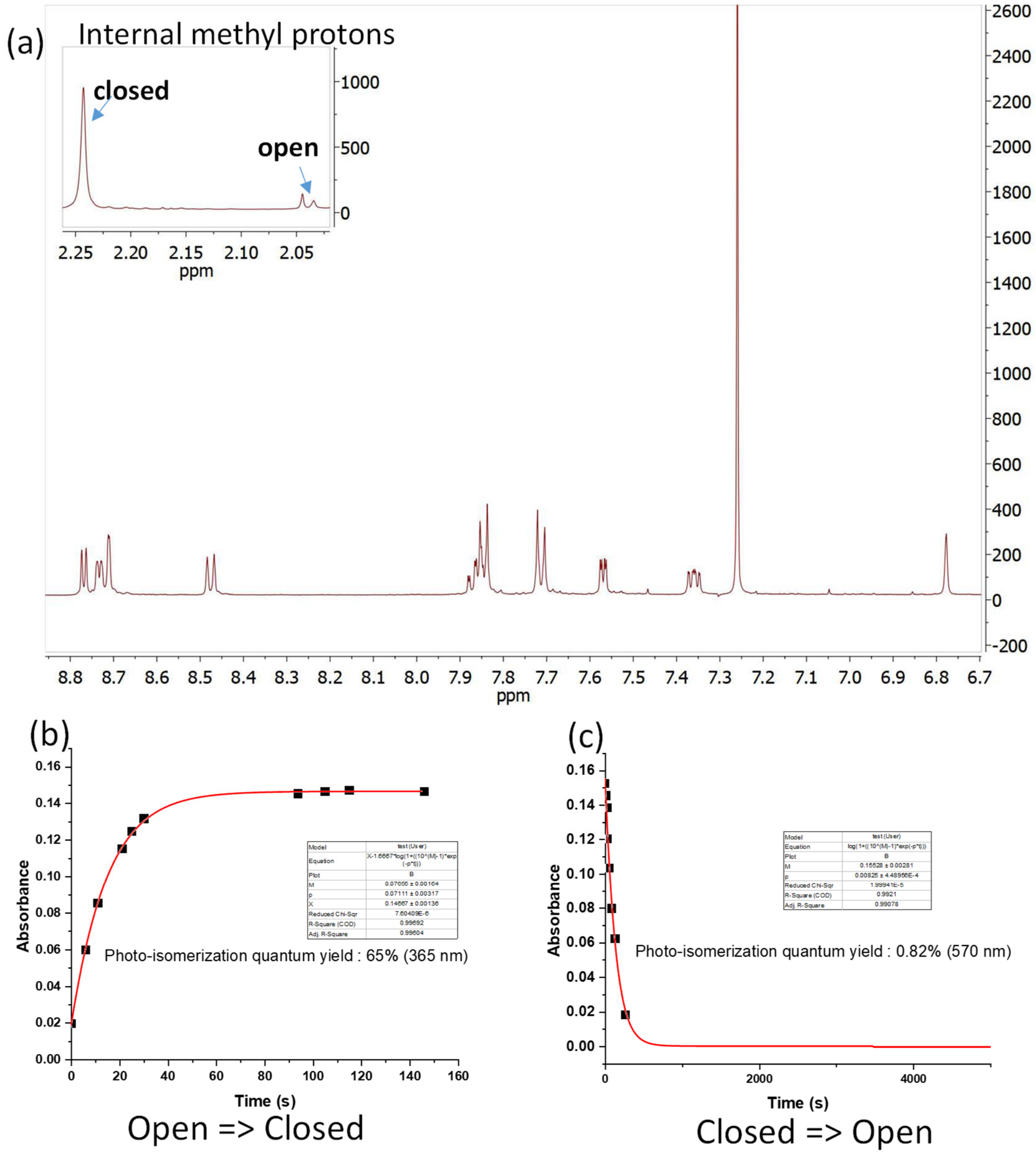

Figure S4: (a) NMR spectrum of the open and the closed forms respectively. (b, c) Photo isomerization quantum yield.

As determined from the 1H NMR spectrum of the open form after UV light irradiation, a 96% photo conversion of the open form to the closed form is observed.

3. Calculation

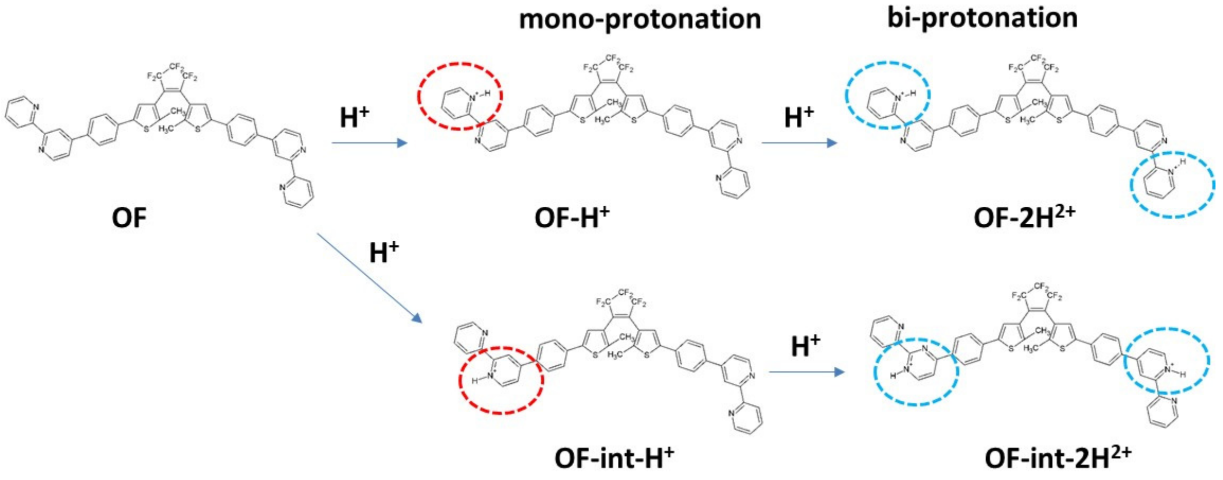

Figure S5. Definition of isomers with different possibilities of mono- or bi- protonation at different bipyridine sites. The scheme uses OF isomers as examples. The CF isomers follow the same rule of definition which will be used for the following calculation.

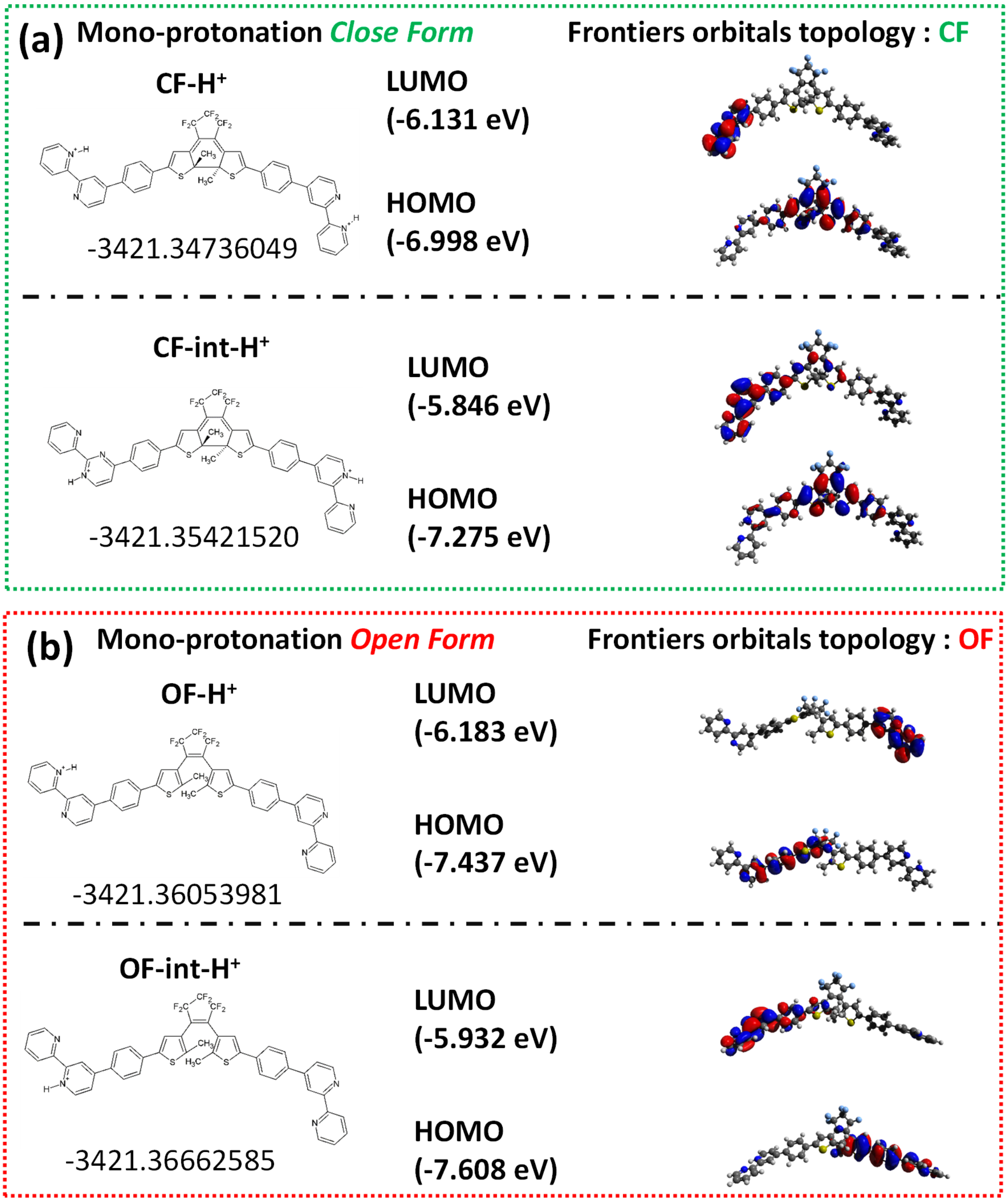

Figure S6. Calculated HOMO LUMO energy levels and orbitals from mono-protonated CF (a) and OF (b) isomers.

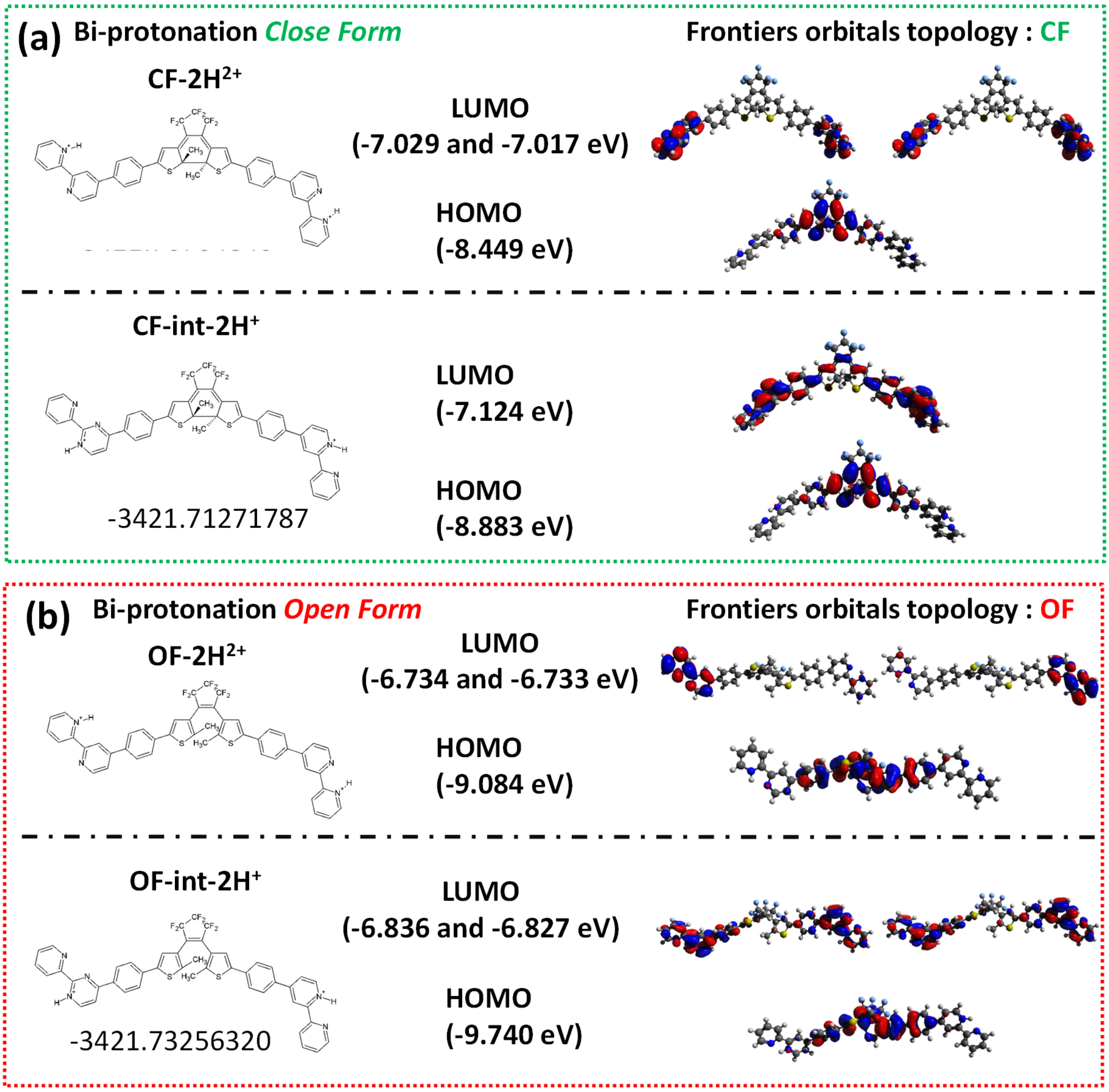

Figure S7. Calculated HOMO LUMO energy levels and orbitals from bi-protonated CF (a) and OF (b) isomers.

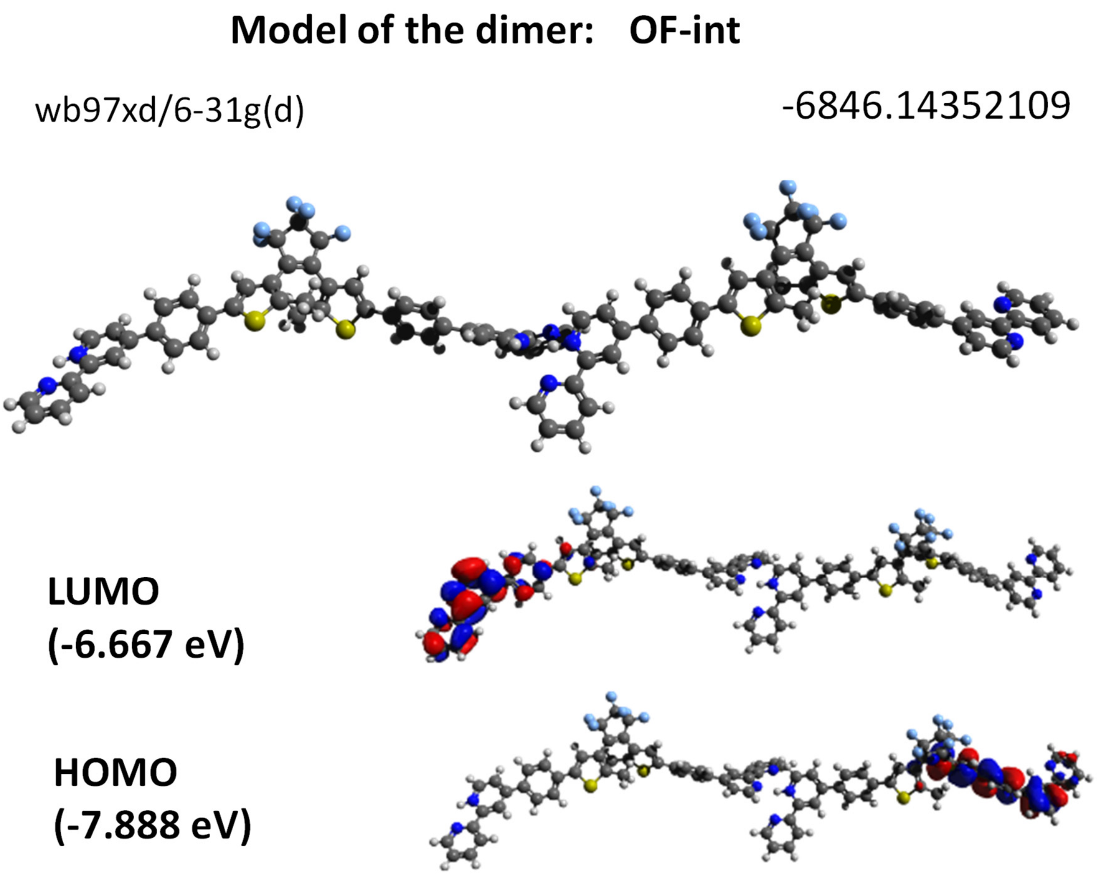

Figure S8. Calculated HOMO LUMO energy levels and orbitals from OF dimers, where the two OF isomers interact through N-H···N hydrogen bond. The H+ is from the protonation of the bipyridine.

The orbitals are observed to be located mainly on the left bpy-DAE-bpy OF isomer bipyridine unit from the LUMO. Whereas for the HOMO, the orbital appears mainly on the right OF isomer, and it localizes on the right arm of the molecular backbone as shown in Figure S6. The calculation orbitals, which we here use the OF state as an example, indicate a clear distinction between the HOMO and the LUMO orbital topographies. On another hand, the dimers model is closer to the environment of the molecules inside the self-organizations than that of a single molecule in the gas phase. The trimer calculation, especially the calculated middle molecules

inside the trimers (molecule B in main text Figure 5a), can give a much better simulation. The calculated results of the trimers are shown in Figure 5.

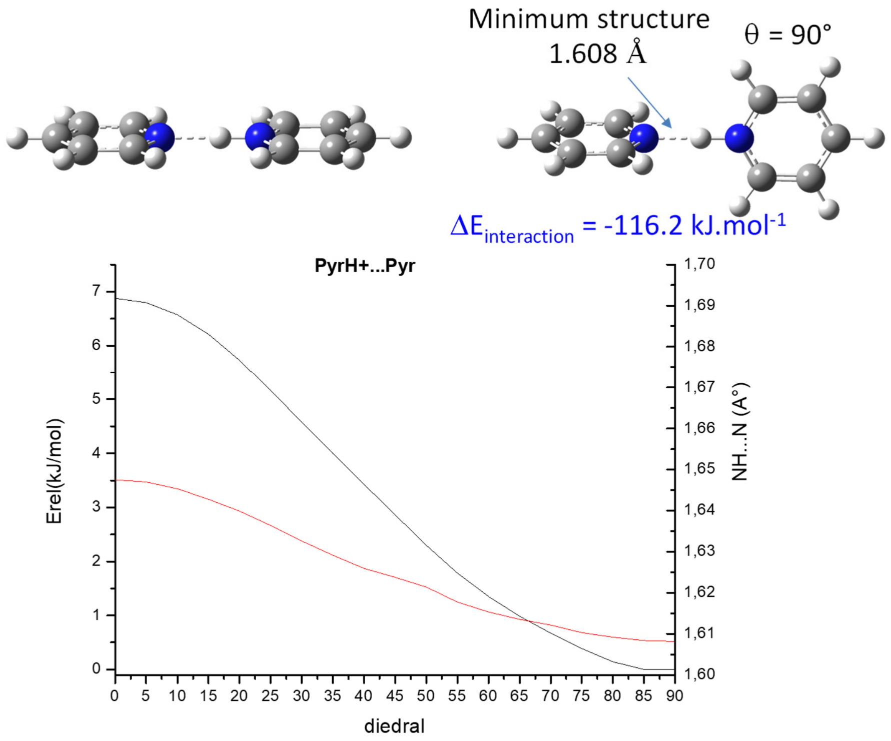

Figure S9. Calculated N-H···N interaction in PyrH$^+$···Pyr dimers. Black curve: the Energy curve for rotation (Θ) between pyridine units when involving the hydrogen bonds. Red curve: hydrogen bond distance N-H···N.

The energy need to rotate the two Pyridine rings in the PyrH$^+$···Pyr dimer from the 90° orientation (more stable) to the planar one is 6.9 kJ.mol^{-1}. This energy value is small which indicates that once absorbed on surface, the two pyridine rings can easily rotate to stabilize the surface organization.

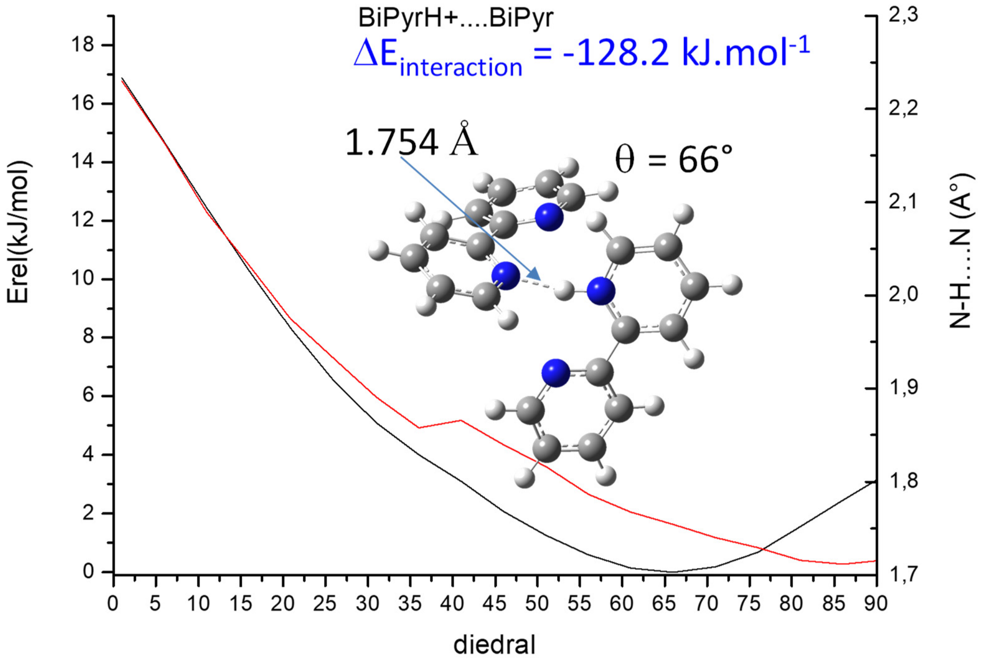

Figure S10. Calculated N-H···N interaction in biPyrH^{+}···biPyr dimers. Black curve: the Energy curve for rotation (Θ) between pyridine units when involving the hydrogen bonds. Red curve: hydrogen bond distance N-H···N.

The more stable orientation in biPyrH+···biPyr dimer is not found at 90° but 66°. The energy penalties to rotate at 90° and 0° are 3.2 kJ.mol and 16.9 kJ.mol, respectively. Therefore the presence of ortho pyridine strongly limits the rotation. However in the most stable structure, the orientation of rings is the result of a balance between the establishment of the hydrogen bond and the small H-H van der waals attraction in the ortho pyridine. As a consequence the interaction energy between the two bipyridine is stronger than that between the two pyridine rings in Figure S6. (The N-H···N hydrogen bond is shorter at 90° orientation but the H···H repulsion destabilizes the structure).

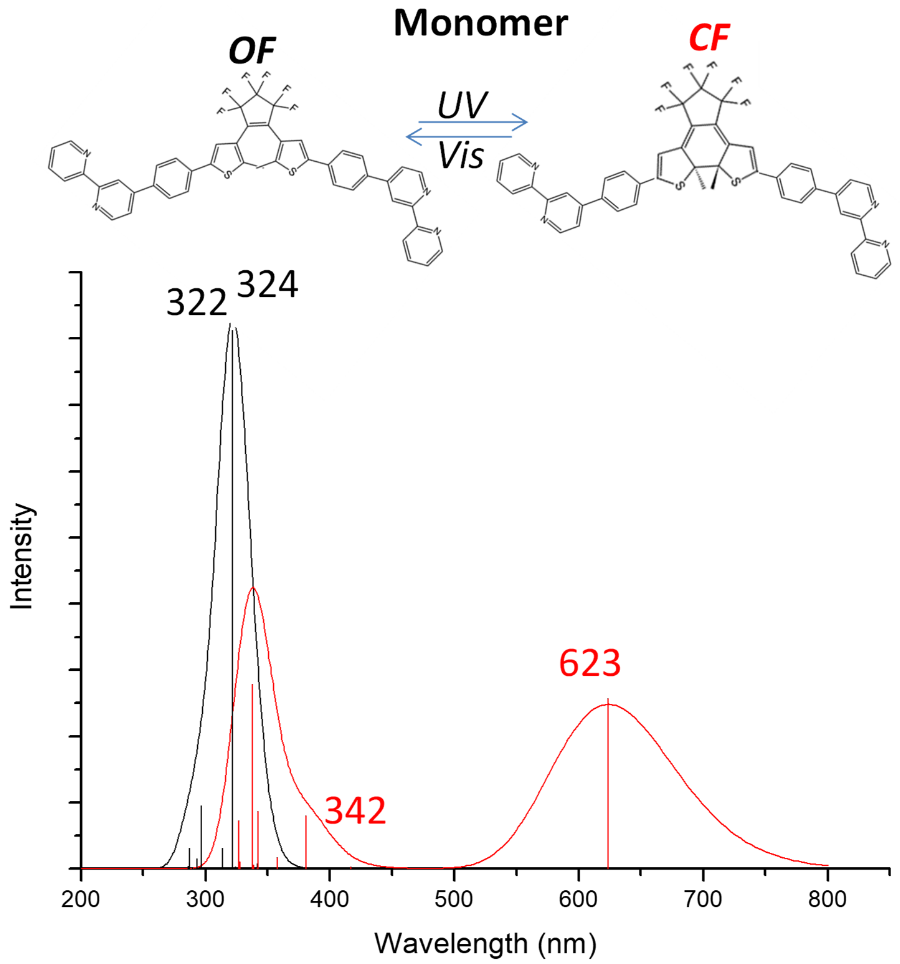

Figure S11. DFT calculated UV-vis spectroscopies of monomer bpy-DAE-bpy in its OF (black) and CF (red) states respectively.

The OF and CF isomers of bpy-DAE-bpy show different trends in the optical spectrums as calculated by the density of functional theory in Figure S9. The OF isomer exhibits an intense optical band around 340 nm which disappears in the spectrum of the CF isomer. The CF isomer yields a higher energy at around 623 nm. These calculated results show close similarity with the experiment UV-vis spectroscopic results In Figure 3b in the main text.
